# A SYSTEMATIC APPROACH TO SPREADING THE WORD ABOUT GERONTOLOGY TRAINING OPPORTUNITIES IN A UNIVERSITY SETTING

**DOI:** 10.1093/geroni/igz038.2287

**Published:** 2019-11-08

**Authors:** Jeremy B Yorgason

**Affiliations:** Brigham Young University, Provo, Utah, United States

## Abstract

Training university students to work in professional gerontology settings is extremely important during an era when the number of older adults is increasing due to the Baby Boom cohort entering their later years. Efforts to reach students are critical given budget and enrollment challenges. Some university students find gerontology resources and training on their own, yet gerontology programs can do much to help students know of opportunities. In this paper, I will share methods that the gerontology program at my university has used to reach out to students and faculty across campus to encourage students to study gerontology. In the last 3 years, student enrollment in this gerontology minor has grown from 65 students housed in 3 colleges, to 275 students housed in 7 colleges. Faculty involvement has grown from a 7-faculty committee, to 61 faculty affiliates. The roles of university resources and fundraising will also be discussed.

